# Alcohol use, risk of suicide, and access to firearms among youth in Colorado

**DOI:** 10.1080/28324765.2025.2607769

**Published:** 2025-12-28

**Authors:** Leslie M. Barnard, Sophie Rosenberg, Ashley Brooks-Russell, Marian E. Betz, Joseph P. Schacht

**Affiliations:** a Department of Epidemiology, Colorado School of Public Health, Aurora, USA; b University of Colorado School of Medicine, Firearm Injury Prevention Initiative, Aurora, USA; c Colorado School of Public Health, Injury and Violence Prevention Center, Aurora, USA; d Department of Biostatistics, Colorado School of Public Health, Aurora, USA; e Department of Psychiatry, University of Colorado School of Medicine, Aurora, USA

**Keywords:** Alcohol, youth, firearm, suicide, mental health

## Abstract

Firearms are the leading cause of death for U.S. children under 18 years of age, driven by their use in suicide attempts. Alcohol use is a risk factor for suicide, and approximately 22% of U.S. high school students reported recent alcohol use. We used data from a population-based health survey of high school students in Colorado, weighted to be representative of public high school students, to examine the association of alcohol use and suicide risk, and access to firearms using weighted frequencies and Rao–Scott chi–square tests. One-fifth (20.3%) of the students reported drinking alcohol in the past 30 days. Compared to non-drinkers, students who reported drinking in the past 30 days were significantly more likely to report that they seriously considered attempting suicide (20.4% vs 8.9%, *p* < 0.001), made a plan to attempt suicide (17.0% vs 7.4%, *p* < 0.001), had actually attempted suicide (11.1% vs 3.9%, *p* < 0.001) and indicated that they could obtain and be ready to fire a loaded gun, regardless of adults' permission (29.0% vs 16.9%, *p* < 0.001). Given the distinct and combined risks of alcohol use and firearm access, the intersection is an important point for suicide prevention. Parents and guardians of youth may constitute an important population for means safety training.

## Introduction

In 2023, firearms were the leading cause of death among children and adolescents under the age of 18 in the United States, surpassing motor vehicle accidents and other causes of injury-related death (Centers for Disease Control and Prevention, 2025a). Suicide accounted for the largest portion of firearm deaths, at 58% of these deaths (Centers for Disease Control and Prevention, 2025a). Limited research exists on youth firearm access, yet there is emerging evidence to suggest concern. Two studies using population-based samples from the U.S. have shown that over one-third of high schoolers have access to firearms (McCarthy et al., 2023) and that six percent of middle and high school students have recently carried handguns (Wright-Kelly et al., 2025). Thus, access to firearms is not uncommon among adolescents.

Simultaneously, alcohol use remains prevalent among youth, with approximately 22% of U.S. high school students reporting alcohol consumption in the past 30 days (Centers for Disease Control and Prevention, 2025b), and approximately 60% of those who currently drank also reported binge drinking (Esser, 2017), which has been relatively steady for the past decade. Prior research with adults has established that both firearm access (Anglemyer et al., 2014; Azad et al., 2020; Dempsey et al., 2019) and alcohol use (Conner et al., 2014; Lange et al., 2023) are independently associated with increased risk for firearm suicide, and when combined, may significantly elevate the potential for fatal outcomes (Morgan et al., 2019). One study found a dose‒response effect between blood alcohol content and the probability of firearm-involved suicide, underscoring the relevance of high-intensity drinking patterns for safety and suicide risk (Lange et al., 2023). Additionally, a well-established body of research links alcohol use to impaired judgment, increased impulsivity and elevated suicide risk (Koller et al., 2002). However, there is more limited research with adolescents. Retrospective findings have shown that in adolescents, nearly half of suicide decedents are reported as having a substance use disorder, predominantly alcohol dependence (Runeson, 1990). One study found that, 47% of adolescent suicide decedents and 78% who died by firearm suicide tested positive for recreational drugs (Vieweg et al., 2006). However, far less is known about how these risk factors interact in adolescent populations.

Adolescence is a critical developmental period marked by increased risk-taking behavior and emotional instability (Chartier et al., 2010; Steinberg, 2010). Youth who consume alcohol may be particularly vulnerable to negative outcomes from risk taking as alcohol can amplify emotional dysregulation and reduce inhibition, which may increase the likelihood of impulsive decisions, including suicidal ideation and self-harm (Esposito-Smythers & Spirito, 2004). Alcohol use, suicidal behavior and firearm access may co-occur in adolescents, highlighting a complex interplay of risk factors that may contribute to heightened vulnerability. Prior surveillance has shown that between 8% and 10% of youth have attempted suicide in the past year (Centers for Disease Control and Prevention, 2022). One study examining youth firearm decedents found that a significant proportion had recently consumed alcohol at the time of death, and many had access to unsecured (unlocked and loaded) firearms in the home (Grossman et al., 2005). Despite the severity of these risks, it remains unclear how many adolescents currently consume alcohol, experience suicidal thoughts and behaviors and also have access to unsecured firearms.

To address this gap, we used data from a population-based survey of Colorado high school students to examine patterns of alcohol use and their association with suicide risk indicators and firearm access. Colorado provides an informative context for this work. The state consistently reports higher-than-national-average levels of firearm ownership (Siegel & Rothman, 2016), historical permissive firearm policies with only recent efforts to strengthen gun laws (Colorado, 2025) which shape adolescents' exposure to lethal means. At the same time, Colorado has faced persistently high rates of youth mental health concerns (Colorado Department of Public Health and Environment, n.d.). The convergence of significant behavioral health challenges and ready access to firearms makes Colorado a critical setting for understanding how environmental and behavioral risk factors interact to heighten vulnerability among adolescents. By analyzing these relationships, we aim to better understand how behavioral health and environmental risk factors elevate vulnerability among adolescents and inform suicide prevention strategies.

## Methods

### Data collection and measurements

The Healthy Kids Colorado Survey (HKCS) is a population-based survey of 9–12th-grade students in Colorado administered online, in classrooms in English or Spanish, in the fall of odd-numbered years (Colorado Department of Public Health and Environment, n.d.). Schools are randomly selected, across 21 regional strata, to be included in the state sample to represent students in grades 9–12. In 2023, the state sample included 49,989 students (RR 64%) at 84 high schools (RR 58%). Participation is voluntary and parents provide consent based on school procedures (either active consent, or a waiver or written consent), and youth provide assent. Teachers administer the survey in a regular class period and are instructed to maintain quiet and avoid walking around the room or answering questions about the survey.

The survey includes questions on the health and well-being of youth and is used to inform the creation of programming to support student success, provide direction for schools and communities to address health issues, and share relevant topics with parents to help them talk to their children about their health and well-being. This includes specific questions on protective factors, mental health, sexual health, tobacco and marijuana use, motor vehicle safety, and body image. Pertinent to this analysis are questions on suicide, alcohol us,e and firearm access.

Although prior research suggests that adolescents, including high-risk youth, generally provide reliable responses to self-report surveys (Pechorro et al., 2019; Ridge et al., 2009), steps were taken to reduce the influence of potentially invalid or extreme responses. Specifically, a subset of respondents were removed based on their responses to another high-risk activity question not included in the primary analysis. This question asked whether participants had ever used inhalants (e.g. glue, aerosol spray cans, paint), cocaine, ecstasy, fentanyl, hemp-derived intoxicating cannabinoids (e.g. Delta-8 and Delta-10 THC), heroin, kratom, methamphetamines, psychedelics and non-prescribed or misused prescription stimulants. Respondents who answered ‘yes’ to all drug categories were excluded from the analytic sample. While this approach may have inadvertently excluded a small sample of high-risk students who responded accurately, it was deemed necessary to minimize the inclusion of systematically problematic or exaggerated responses that could unduly influence results. Given the absence of standardized protocols for identifying such responses in adolescent survey research, this method provides a conservative strategy for enhancing data quality. A total of 217 responses were excluded in total with this approach, leaving an analytical sample of 49,772 unweighted survey responses. Sensitivity analysis examining the effect of removal of extreme responses can be found in Appendix C.

This study was deemed exempt by the Colorado Multiple Institutional Review Board (protocol #06-0397).

### Statistical approach

Survey weights were applied to represent the underlying population of student enrollment in all Colorado public high schools. These weights accounted for sampling design, school and student nonparticipation and nonresponse, and differences in grade, gender, and race and ethnicity between the sample and the population.

All data were assessed for missing and/or implausible values using frequency tables prior to formal analysis. Variables were then re-categorized, and bivariate associations of demographic variables, potential confounders, exposures, and outcomes were tested using two-sample *t*-tests for continuous variables and X^2^ tests for categorical variables. All variables with greater than 10% missing were assessed for patterns in missingness by our main outcomes and exposures of interest (Appendix). This analysis was used for the interpretation of potential bias or influence of missing data on produced odds ratios.

First, we used survey-weighted frequencies to describe how many high school students in Colorado currently drink alcohol, defined as those who reported drinking at least 1 drink of alcohol in the last 30 days. Next, we assessed differences among those who reported currently drinking vs not using survey-weighted Rao‒Scott Χ^2^ tests. Finally, we used complete case analysis to produce odds and adjusted odds of suicide risk indicators (seriously considered attempting suicide, made a suicide plan, actually attempted suicide in the past year) and ease of firearm access (as assessed by the question, ‘Could you get and be ready to fire a loaded gun without a parent or other adult's permission?’). Confounders in models included the following: grade, gender identity, sexual identity, race, mothers' education, physical disabilities or long-term health problems, and emotional problems or learning disabilities were included based on prior literature and theory. All analyses were performed in SAS version 9.4.

## Results

The 2023 sample included 49,772 unweighted and 239,541 weighted responses. The largest portion of students were male (49.5%), heterosexual and cisgender (78.6%), White (52.3%), and non-Hispanic (80.2%), and 15–17 years old (73.4%, Table 1). One-fifth (20.3%) of students reported drinking at least one drink of alcohol in the past 30 days, of whom most drank on 1–2 days (60.2%), followed by 3–5 days (20.9%), and less than 20% indicated they drank on more than 5 days in the last 30 days. Over half (57.6%) of those who drank in the past 30 days reported having 4 or more drinks consecutively, near or above the threshold for binge drinking in youth (Chung et al., 2018). Overall, of those who drank in the past 30 days, 20.5% reported they seriously considered attempting suicide, 17.3% made a plan about how they would attempt suicide, 11.1% reported they actually attempted suicide, and 31.0% reported they could get and be ready to fire a loaded gun without permission.

**Table 1.** Demographic factors among final sample of Healthy Kids Colorado 2023 survey, by current drinking status.

	Total (*n* (%), (unweighted *n* = 49,772, weighted *n* = 239,541)	During the past 30 days, on how many days did you have at least one drink of alcohol?		
		1–30 days (*n* (%), unweighted *n* = 9197, weighted *n* = 43,055)	0 days (*n* (%), unweighted *n* = 35,308, weighted *n* = 169,071)	*p*-value
Grade				<0.001
9th grade	14,705 (29.8)	1701 (18.6)	11,551 (32.9)	
10th grade	13,484 (27.3)	2209 (24.1)	9938 (28.3)	
11th grade	11,624 (23.6)	2532 (27.7)	7918 (22.6)	
12th grade	2590 (19.3)	2708 (29.6)	5706 (16.3)	
Gender				<0.001
Female	22,678 (46.4)	4745 (52.0)	15,891 (45.3)	
Male	24,163 (49.5)	4026 (44.1)	17,744 (50.6)	
Nonbinary/another identity/not sure	2016 (4.1)	351 (3.8)	1443 (4.1)	
During the past 30 days, on how many days did you have 4 or more drinks of alcohol in a row?^*^				**–**
Yes	5125 (11.8)	5116 (57.6)	**–**	
No	38,365 (88.2)	3761 (42.4)	**–**	
During the past 30 days, on how many days did you have at least one drink of alcohol?^*^				**–**
1 or 2 days	5537 (60.2)	5537 (60.2)	**–**	
3 to 5 days	1919 (20.9)	1919 (20.9)	**–**	
6 to 9 days	889 (9.7)	889 (9.7)	**–**	
10 + days	852 (9.3)	852 (9.3)	**–**	
Seriously considered attempting suicide in the past year				<0.001
Yes	5240 (11.5)	1865 (20.5)	3220 (9.2)	
No	40,379 (88.5)	7248 (79.5)	31,629 (90.8)	
Made a plan about how you would attempt suicide in the past year				<0.001
Yes	4371 (9.6)	1575 (17.3)	2662 (7.7)	
No	41,159 (90.4)	7518 (82.7)	32,128 (92.3)	
Actually attempted suicide in the past year				<0.001
Yes	2453 (5.4)	1013 (11.1)	1343 (3.9)	
No	43,161 (94.6)	8087 (88.9)	33,539 (96.1)	
Could get and be ready to fire a loaded gun without a parent or other adult's permission				<0.001
Yes	8373 (21.4)	2446 (31.0)	5860 (19.0)	
No	30,734 (78.6)	5436 (68.0)	25,055 (81.0)	

* Only among those who reported currently drinking (during the past 30 days reported having at least one drink of alcohol).

Significant demographic and behavioral differences were observed between students who reported current drinking and those who did not (Table 1). Those who reported current drinking were more likely to be in a higher grade level (29.6% in 12th grade vs 16.3%, *p* < .001), female (52.0% vs 45.3%, *p* < .001), LGBTQ + (23.4% vs 20.9%, *p* < .001), White (57.3% vs 51.6%, *p* < .001), say that they always/most of the time felt that they were able to talk to a friend about their feelings (58.1% vs 59.6%, *p* < .001), and have a physical or emotional disability (29.8% vs 21.2%, *p* < .001) compared to those who were not currently drinking (Table 1).

Among those who reported drinking alcohol in the past 30 days, 20.5% of students reported they seriously considered attempting suicide in the last year, 17.3% made a plan to attempt suicide in the last year, 11.1% actually attempted suicide in the last year and 31.0% indicated that they could currently get a loaded gun, regardless of adult's permission (Table 1). A prevalence of 1.4% of students reported they drank alcohol in the past 30 days, had seriously considered attempting suicide in the past year, and could get and be ready to fire a loaded gun without a parent or other adult's permission.

Prior to adjusting for confounders, crude odds ratios were all significant at the 0.05 level. Students who reported drinking alcohol in the past 30 days had 2.56 (95% CI: 2.41–2.71) times the odds of reporting they had seriously considered attempting suicide, 2.48 (95% CI: 2.20–2.79) times the odds of reporting they had made a plan about how they would attempt suicide, 2.86 (95% CI: 2.59–3.17) times the odds of reporting they had actually attempted suicide and 1.96 (95% CI: 1.75–2.19) times the odds of reporting they could get and be ready to fire a loaded gun without a parent or adult's permission, compared to those who did not currently drink alcohol. After adjusting for confounders, students who reported drinking alcohol in the past 30 days had 2.35 (95% CI: 2.15–2.56) times the odds of reporting they had seriously considered attempting suicide, 2.20 (95% CI: 1.96–2.48) time times the odds of reporting they had made a plan about how they would attempt suicide, 2.71 (95% CI: 2.39–3.07) times the odds of reporting they had actually attempted suicide and 1.85 (95% CI: 1.66–2.07) times the odds of reporting they could get and be ready to fire a loaded gun without a parent or adult's permission, compared to those who did not currently drink alcohol (*p* < 0.001 for all differences, Table 2).

**Table 2.** Adjusted and unadjusted odds ratios for suicide risk factors, firearm access, and current drinking status.

	Unadjusted model		Adjusted model^*^	
	OR (95% CI)	*p*-value	aOR (95% CI)	*p*-value
Seriously considered attempting suicide (*N* = 43,962, aN = 36,452)	2.56 (2.41, 2.71)	<0.001	2.35 (2.15, 2.56)	<0.001
Made a plan about how you would attempt suicide (*N* = 43,883 aN = 36,402)	2.48 (2.20, 2.79)	<0.001	2.20 (1.96, 2.48)	<0.001
Actually attempted suicide (*N* = 43,982 aN = 36,472)	2.86 (2.59, 3.17)	<0.001	2.71 (2.39, 3.07)	<0.001
Could get and be ready to fire a loaded gun without a parent or other adult's permission (*N* = 38,797 aN = 32,552)	1.96 (1.75, 2.19)	<0.001	1.85 (1.66, 2.07)	<0.001

* Adjusted for: grade, gender identity, LGBTQ, race, mothers' education, have a physical or emotional disability.

## Discussion

In this population-based study of Colorado high school students, we found that current alcohol use was associated with increased odds of suicide-related thoughts and behaviors, as well as perceived ease of access to firearms. Specifically, youth who reported drinking alcohol in the past 30 days had more than twice the odds of seriously considering suicide, planning a suicide attempt, or reporting an actual attempt, compared to their non-drinking peers. Additionally, they were significantly more likely to report being able to access and fire a loaded gun without adult permission. These differences persisted even after adjusting for a range of potential confounding factors, including demographic characteristics and physical or emotional disabilities, suggesting that the associations are robust.

Our results are consistent with prior research in adult populations demonstrating that alcohol use is an independent risk factor for suicide and firearm-related injury (Conner et al., 2014; Lange et al., 2023; Morgan et al., 2019), and extends this association to adolescents. We found that youth who drank had over two times the odds of reporting they had considered attempting suicide, had made a plan about how they would attempt suicide, had attempted suicide and had access to firearms, compared to those who did not currently drink alcohol. These results are consistent with adult populations, in which 5% of past-month binge drinkers reported serious thoughts of suicide in the U.S. (Substance Abuse and Mental Health Services Administration, 2025) and in two state, binge and chronic alcohol use were somewhat more prevalent among adults from firearm-owning households (PR = 1.1 and1.2) (Morgan et al., 2018; Barnard et al., 2025). These results also align with previous work indicating that firearm access among youth is not uncommon. This study adds that alcohol-using youth may be particularly likely to report ease of firearm access. These findings highlight a concerning intersection of alcohol use, suicide risk and firearm access among adolescents. Adolescence is marked by emotional reactivity, impulsivity and risk-taking, which may amplify alcohol's impact on decision-making and increase the risk of self-harm. This is concerning, given the well-established lethality of firearms (Shenassa et al., 2003) in suicide attempts and the known disinhibiting effects of alcohol.

The co-occurrence of alcohol use, suicidal thoughts and behaviors and perceived access to firearms highlights a complex and potentially deadly intersection of risk factors. Although only a small percentage of students (1.4%) reported all three (current drinking, seriously considered suicide and ease of firearm access) this subgroup represents a critically vulnerable population at markedly increased risk for lethal self-harm. Each of these issues independently increases the risk of harm, but when present together, they may compound one another, amplifying the likelihood of impulsive, high-lethality suicide attempts. There may be other confounding variables associated with both alcohol use and access to firearms such as lack of parental supervision which may be targeted to prevent these especially high-risk groups. The observation that over half of students who drank reported binge drinking, which is associated with greater acute health harms than lower levels of drinking (Chung et al., 2018), further underscores the potential for impaired judgment and heightened risk.

This study is not without limitations. First, these data are cross-sectional, limiting our ability to understand the directionality of relationships identified. Alcohol use may act as a proxy for broader psychosocial risk factors such as peer influence, household dysfunction, or adverse childhood experiences, all of which may also relate to unsafe firearm storage. Second, all measures were self-reported and may be subject to recall bias or social desirability bias, although prior research supports the general validity of adolescent self-report surveys (Runeson, 1990; Vieweg et al., 2006). Perceived firearm access may not fully capture the nuances of real-world firearm availability. Missing data did exist. Among our outcomes of interest, the highest level of missingness was the firearm access variable with 24% missing data. In our analysis of missingness, we found that race/ethnicity was the only variable with missingness associated with all suicide outcomes examined. Among all suicide outcomes examined, more missing data existed among black students, therefore potentially biasing our result. Black individuals reported lower levels of drinking compared to non-black individuals; therefore, our odds and adjusted odds ratios may be inflated as a result of this missing data. However, as Black students represented only 3.3% of our weighted sample, any bias is likely minimal. Nearly all covariates with missingness were associated with our firearm access variable. Students who were male, in higher grade levels, identified as heterosexual and cisgender, were Black, or whose mothers had a high school education or less were more likely to have missing data on firearm access (all between 16% and 25% missing). We also included an analysis comparing weighted prevalence estimates before and after case-wise deletion (Appendix D), which revealed differences between missing and non-missing data, suggesting these data are missing not at random. As a result, findings related to firearm access may not be fully generalizable to these subgroups and should be interpreted with caution. Finally, we excluded a small number of respondents with extreme patterns of drug use to improve data reliability. This may have resulted in the omission of some of the highest-risk youth, although it was less than 0.5% of the sample and sensitivity analysis (Appendix C) showed no impact on direction of associations and statistical significance.

Future research is needed to understand the intersection between alcohol use and other substances, which we did not explore in this study. Additionally, the finding that over half of youth who drank reported consuming four or more drinks in a row may indicate that binge/heavy episodic alcohol use compounds these risks and should be explored, especially in the context of mental health, suicidal ideation and firearm access. Additionally, measures on increasing levels of alcohol intake should be added to this survey so that a dose‒response can be detected. Finally, future research should focus on longitudinal studies to better understand the causal pathways linking these factors and to identify effective intervention points to reduce risk among this vulnerable population.

These findings emphasize the need for integrated prevention efforts that address alcohol use, mental health and firearm safety concurrently. Suicide prevention efforts targeting youth may integrate screening and interventions for alcohol use as a modifiable risk factor. Strategies to reduce youth access to firearms and alcohol, such as safe storage campaigns and firearm safety education for parents, remain a necessary component of comprehensive suicide prevention (Miller et al., 2020). Beyond parents, schools and communities may benefit from programming that jointly addresses alcohol use, mental health and firearm access. Clinicians should also be aware of the elevated risks associated with alcohol use when conducting mental health assessments or safety planning with adolescents.

There are several policy implications for these findings. Colorado's safe storage law prohibits firearm owners from storing their firearms so that children/youth can access them. Previous research has shown that among states with similar laws, many firearm owners are unaware of these laws (Miller et al., 2022). In combination with our findings, this suggests the need for communication campaigns and dissemination and implementation research to ensure all Colorado firearm owners are aware of and abide by this law and therefore prevent youth firearm access in their homes and cars. This is especially important for the 1.4% of students who reported current drinking, seriously considered suicide and ease of firearm access. Given the co-occurrence of these risks, multi-level approaches that involve families, schools, healthcare systems and policies are warranted to identify the highest risk individuals and ensure their safety.

In conclusion, this study provides important evidence that alcohol use among high school students is strongly associated with suicide risk and ease of firearm access. These findings underscore the need for integrated prevention strategies for adolescents to reduce the likelihood of fatal outcomes and support safer, healthier youth development.

## Supplementary Material

HKCO Appendix A B C and D.docx HKCO Appendix A B C and D.docx

## Data Availability

The data that support the findings of this study are available on request from the corresponding author, LB.
