# Supplementary material for: Alcohol use, risk of suicide, and access to firearms among youth in Colorado
Source: Cogent Ment Health. 2025 Dec 28;5(1):2607769. doi: 10.1080/28324765.2025.2607769 (PMC12777784; doi:10.1080/28324765.2025.2607769)
Supplement: HKCO Appendix A B C and D.docx [file OAMH_A_2607769_SM0117.docx]

**Appendix A: Supplementary demographic factors among final sample of Healthy Kids Colorado 2023 survey, by current drinking status**

|  | Total (n (%), (unweighted  n = 49,772, weighted n=239,541) | **During the past 30 days, on how many days did you have at least one drink of alcohol?** | | |
| --- | --- | --- | --- | --- |
|  |  | **1-30 days** (n (%), unweighted n = 9,197, weighted n=43,055) | **0 days** (n (%), unweighted n = 35,308, weighted n=169,071) | p-value |
| **Age** |  |  |  | <0.001 |
| 14 years old or younger | 10393 (21.0) | 1109 (12.1) | 8272 (23.5) |  |
| 15-17 years old | 36386 (73.4) | 7266 (79.1) | 25374 (72.0) |  |
| 18 years old or older | 2821 (5.7) | 814 (8.9) | 1607 (4.6) |  |
| **LGBTQ+** |  |  |  | <0.001 |
| Heterosexual & Cisgender | 37083 (78.6) | 6876 (76.6) | 26919 (79.1) |  |
| LGBTQ+ | 10077 (21.4) | 2105 (23.4) | 7132 (20.9) |  |
| **Race/Ethnicity** |  |  |  | <0.001 |
| White | 25081 (52.3) | 5220 (57.3) | 17959 (51.6) |  |
| Black | 1601 (3.3) | 155 (1.7) | 1241 (3.6) |  |
| Hispanic | 9510 (19.8) | 1545 (17.0) | 7052 (20.3) |  |
| Another identity/Multiracial | 11794 (24.6) | 2185 (24.0) | 8544 (24.5) |  |
| **During your life, how often have you felt that you were able to talk to a friend about your feelings?** |  |  |  | <0.001 |
| Always/Most of the time | 27094 (59.3) | 5311 (58.1) | 20891 (59.6) |  |
| Sometimes | 9868 (21.6) | 2125 (23.2) | 7417 (21.2) |  |
| Rarely/Never | 8735 (19.1) | 1706 (18.7) | 6712 (19.2) |  |
| **Mother’s Education** |  |  |  | 0.165 |
| High school or less | 12190 (30.2) | 2478 (29.3) | 9039 (30.1) |  |
| Some college | 5049 (12.5) | 1042 (12.3) | 3764 (12.6) |  |
| College or graduate | 23140 (57.3) | 4949 (58.4) | 17174 (57.3) |  |
| **Have a physical or emotional disability** |  |  |  | <0.001 |
| **Yes** | 10753 (23.0) | 2734 (29.8) | 7472 (21.2) |  |
| **No** | 36000 (77.0) | 6441 (70.2) | 27710 (78.8) |  |

*Only among those who reported currently drinking (During the past 30 days reported having at least one drink of alcohol)

**Appendix B: Analysis of Missing Data**

| **Seriously considered attempting suicide (% missing)** | | | | | | |  |
| --- | --- | --- | --- | --- | --- | --- | --- |
|  | Missing | | | Not missing | p-value | |  |
| **Grade** | | | | | | |  |
| 9^th^ Grade | 1159 (8.9%) | | | 13546 (91.1%) | 0.245 | |  |
| 10^th^ Grade | 1073 (8.4%) | | | 12411 (91.6%) |  |  |  |
| 11^th^ Grade | 911 (7.9%) | | | 10713 (92.1%) |  |  |  |
| 12^th^ Grade | 828 (9.3%) | | | 8692 (90.7%) |  |  |  |
| **Gender** | | | | | | |  |
| Female | 1515 (7.6%) | | | 21163 (92.4%) | 0.488 | |  |
| Male | 1832 (8.1%) | | | 22331 (91.9%) |  |  |  |
| Nonbinary/Another identity/Not sure | 208 (8.2%) | | | 1808 (91.8%) |  |  |  |
| **LGBTQ+** | | | | | | |  |
| Heterosexual & Cisgender | 2330 (7.0%) | | | 34753 (93.0%) | 0.447 | |  |
| LGBTQ+ | 710 (7.4%) | | | 9367 (92.6%) |  |  |  |
| **Race/Ethnicity** | | | | | | |  |
| White | 1412 (6.0%) | | | 23669 (94.0%) | **<0.001** | |  |
| Black | 151 (10.2%) | | | 1450 (89.8%) |  |  |  |
| Hispanic | 645 (7.6%) | | | 8865 (92.4%) |  |  |  |
| Another identity/Multiracial | 774 (7.7%) | | | 11020 (92.3%) |  |  |  |
| **Mother’s Education** | | | | | | |  |
| High school or less | 371 (3.0%) | | | 11819 (97.0%) | 0.474 | |  |
| Some college | 131 (3.0%) | | | 4918 (97.0%) |  |  |  |
| College or graduate | 530 (2.6%) | | | 22610 (97.4%) |  |  |  |
| **Have a physical or emotional disability** | | | | | | |  |
| Yes | 328 (3.1%) | | | 10425 (96.9%) | 0.442 | |  |
| No | 947 (2.9%) | | | 35353 (97.1%) |  |  |  |
| **Made a plan about how you would attempt suicide (% missing)** | | | | | | | |
|  | | Missing | Not missing | | | p-value | |
| **Grade** | | | | | | | |
| 9^th^ Grade | | 1194 (9.1%) | 13511 (90.9%) | | | 0.156 | |
| 10^th^ Grade | | 1096 (8.6%) | 12388 (91.4%) | | |  |  |
| 11^th^ Grade | | 927 (8.0%) | 10697 (92.0%) | | |  |  |
| 12^th^ Grade | | 842 (9.5%) | 8678 (90.5%) | | |  |  |
| **Gender** | | | | | | | |
| Female | | 1571 (7.7%) | 21107 (92.3%) | | | 0.582 | |
| Male | | 1865 (8.3%) | 22298 (91.7%) | | |  |  |
| Nonbinary/Another identity/Not sure | | 207 (8.2%) | 1809 (91.8%) | | |  |  |
| **LGBTQ+** | | | | | | | |
| Heterosexual & Cisgender | | 2387 (7.1%) | 34696 (92.9%) | | | 0.474 | |
| LGBTQ+ | | 729 (7.5%) | 9348 (92.5%) | | |  |  |
| **Race/Ethnicity** | | | | | | | |
| White | | 1446 (6.1%) | 23635 (93.9%) | | | **0.001** | |
| Black | | 150 (9.9%) | 1451 (90.1%) | | |  |  |
| Hispanic | | 670 (7.8%) | 8840 (92.2%) | | |  |  |
| Another identity/Multiracial | | 805 (8.2%) | 10989 (91.8%) | | |  |  |
| **Mother’s Education** | | | | | | | |
| High school or less | | 378 (3.0%) | 11812 (97.0%) | | | 0.831 | |
| Some college | | 132 (3.1%) | 4917 (96.9%) | | |  |  |
| College or graduate | | 583 (2.9%) | 22557 (97.1%) | | |  |  |
| **Have a physical or emotional disability** | | | | | | | |
| Yes | | 351 (3.3%) | 10402 (96.7%) | | | 0.518 | |
| No | | 1011 (3.1%) | 34989 (96.9%) | | |  |  |
| **Actually attempted suicide (% missing)** | | | | | | | |
|  | | Missing | Not missing | | | p-value | |
| **Grade** | | | | | | | |
| 9^th^ Grade | | 1152 (8.9%) | 13553 (91.1%) | | | 0.217 | |
| 10^th^ Grade | | 1065 (8.3%) | 12419 (91.7%) | | |  |  |
| 11^th^ Grade | | 917 (8.0%) | 10707 (92.0%) | | |  |  |
| 12^th^ Grade | | 843 (9.4%) | 8677 (90.6%) | | |  |  |
| **Gender** | | | | | | | |
| Female | | 1518 (7.6%) | 21160 (92.4%) | | | 0.507 | |
| Male | | 1837 (8.1%) | 22326 (91.9%) | | |  |  |
| Nonbinary/Another identity/Not sure | | 200 (7.6%) | 1816 (92.4%) | | |  |  |
| **LGBTQ+** | | | | | | | |
| **Heterosexual & Cisgender** | | 2337 (7.0%) | 34746 (93.0%) | | | 0.844 | |
| **LGBTQ+** | | 696 (7.1%) | 9381 (92.9%) | | |  |  |
| **Race/Ethnicity** | | | | | | | |
| White | | 1401 (5.9%) | 23680 (94.1%) | | | **<0.001** | |
| Black | | 153 (10.3%) | 1448 (89.7%) | | |  |  |
| Hispanic | | 643 (7.6%) | 8867 (92.4%) | | |  |  |
| Another identity/Multiracial | | 779 (7.8%) | 11015 (92.2%) | | |  |  |
| **Mother’s Education** | | | | | | | |
| High school or less | | 360 (2.9%) | 11830 (97.1%) | | | 0.560 | |
| Some college | | 130 (3.1%) | 4919 (96.9%) | | |  |  |
| College or graduate | | 531 (2.7%) | 22609 (97.3%) | | |  |  |
| **Have a physical or emotional disability** | | | | | | | |
| Yes | | 339 (3.2%) | 10414 (96.8%) | | | 0.262 | |
| No | | 939 (2.9%) | 35061 (97.1%) | | |  |  |
| **Could get and be ready to fire a loaded gun without a parent or other adult’s permission (% missing)** | | | | | | | |
|  | | Missing | Not missing | | | p-value | |
| **Grade** | | | | | | | |
| 9^th^ Grade | | 3170 (25.3%) | 11535 (74.7%) | | | **0.025** | |
| 10^th^ Grade | | 2753 (22.7%) | 10731 (77.3%) | | |  |  |
| 11^th^ Grade | | 2388 (22.3%) | 9236 (77.7%) | | |  |  |
| 12^th^ Grade | | 2109 (25.0%) | 7411 (75.0%) | | |  |  |
| **Gender** | | | | | | | |
| Female | | 4416 (22.6%) | 18262 (77.4%) | | | **0.002** | |
| Male | | 5201 (24.3%) | 18962 (75.7%) | | |  |  |
| Nonbinary/Another identity/Not sure | | 378 (17.3%) | 1638 (82.7%) | | |  |  |
| **LGBTQ+** | | | | | | | |
| Heterosexual & Cisgender | | 7429 (23.0%) | 29654 (77.0%) | | | **0.006** | |
| LGBTQ+ | | 1764 (19.1%) | 8313 (80.9%) | | |  |  |
| **Race/Ethnicity** | | | | | | | |
| White | | 4317 (18.6%) | 20764 (81.4%) | | | **<0.001** | |
| Black | | 464 (30.7%) | 1137 (69.3%) | | |  |  |
| Hispanic | | 2206 (25.8%) | 7304 (74.2%) | | |  |  |
| Another identity/Multiracial | | 2312 (23.5%) | 9482 (76.5%) | | |  |  |
| **Mother’s Education** | | | | | | | |
| High school or less | | 2241 (20.0%) | 9949 (80.0%) | | | **0.002** | |
| Some college | | 717 (16.4%) | 4332 (83.6%) | | |  |  |
| College or graduate | | 3323 (16.6%) | 19817 (83.4%) | | |  |  |
| **Have a physical or emotional disability** | | | | | | | |
| Yes | | 1777 (18.9%) | 8976 (81.1%) | | | 0.814 | |
| No | | 5966 (19.2%) | 30034 (80.8%) | | |  |  |

**Appendix C: Sensitivity Analysis for Extreme Response Exclusion**

| **Prevalence Estimates of suicide risk factors with and without excluded responses** | | | | |
| --- | --- | --- | --- | --- |
|  | % (95% CI) with excluded responses | | % (95% CI) without excluded responses | |
| Seriously considered attempting suicide | 11.1 (10.2, 12.1) | | 11.0 (10.1, 12.0) | |
| Made a plan about how you would attempt suicide | 9.3 (8.6, 9.9) | | 9.1 (8.5, 9.8) | |
| Actually attempted suicide | 5.5 (4.9, 6.0) | | 5.3 (4.8, 5.9) | |
| Could get and be ready to fire a loaded gun without a parent or other adult’s permission | 19.4 (17.9, 21.0) | | 19.3 (17.8, 20.8) | |
| **Unadjusted Odds Ratios for suicide risk factors and firearm access and current drinking status with and without excluded responses** | | | | |
|  | Unadjusted model with excluded responses | | Unadjusted model without excluded responses | |
|  | OR (95% CI) | p-value | OR (95% CI) | p-value |
| Seriously considered attempting suicide | 2.56 (2.41, 2.71) | <0.001 | 2.62 (2.48, 2.77) | <0.001 |
| Made a plan about how you would attempt suicide | 2.48 (2.20, 2.71) | <0.001 | 2.56 (2.28, 2.88) | <0.001 |
| Actually attempted suicide | 2.86 (2.59, 3.17) | <0.001 | 3.04 (2.75, 3.37) | <0.001 |
| Could get and be ready to fire a loaded gun without a parent or other adult’s permission | 1.96 (1.75, 2.19) | <0.001 | 2.00 (1.79, 2.24) | <0.001 |
| **Adjusted Odds Ratios for suicide risk factors and firearm access and current drinking status with and without excluded responses** | | | | |
|  | Adjusted model with excluded responses | | Adjusted model without excluded responses | |
|  | aOR (95% CI) | p-value | aOR (95% CI) | p-value |
| Seriously considered attempting suicide | 2.35 (2.15, 2.56) | <0.001 | 2.37 (2.18, 2.58) | <0.001 |
| Made a plan about how you would attempt suicide | 2.20 (1.96, 2.48) | <0.001 | 2.25 (2.00, 2.52) | <0.001 |
| Actually attempted suicide | 2.71 (2.39, 3.07) | <0.001 | 2.82 (2.50, 3.18) | <0.001 |
| Could get and be ready to fire a loaded gun without a parent or other adult’s permission | 1.85 (1.66, 2.07) | <0.001 | 1.87 (1.68, 2.09) | <0.001 |

**Appendix D: Sensitivity Analysis of Firearm Access Missing Data**

|  |  | %(95% CI) with missing data | %(95% CI) without missing data |
| --- | --- | --- | --- |
| Grafe | 9^th^ Grade | **13.4 (12.3, 14.4)** | **17.9 (16.8, 19.0)** |
|  | 10^th^ Grade | **15.7 (13.9, 17.5)** | **20.3 (18.2, 22.4)** |
|  | 11^th^ Grade | **15.0 (13.4, 16.6)** | **19.3 (17.3, 21.3)** |
|  | 12^th^ Grade | **14.9 (13.2, 16.6)** | **19.9 (17.6, 22.1)** |
|  | Missing | 6.0 (3.8, 8.2) | **-** |
| Gender | Female | **12.3 (11.0, 13.6)** | **15.9 (14.2, 17.6)** |
|  | Male | **17.0 (15.6, 18.4)** | **22.5 (20.8, 24.1)** |
|  | Nonbinary/Another identity/Not sure | 16.7 (13.3, 20.1) | 20.2 (16.8, 23.6) |
|  | Missing | 4.7 (2.9, 6.5) | **-** |
| LGBTQ+ | Heterosexual & Cisgender | **15.4 (14.1, 16.7)** | **20.0 (18.3, 21.6)** |
|  | LGBTQ+ | **13.6 (12.6, 14.6)** | **16.8 (15.6, 18.1)** |
|  | Missing | 7.9 (5.6, 10.2) | **-** |
| Race/Ethnicity | White | **19.3 (18.1, 20.5)** | **23.7 (22.3, 25.2)** |
|  | Black | 8.4 (6.6, 10.1) | 12.1 (10.0, 14.1) |
|  | Hispanic | 9.9 (8.4, 11.3) | 13.3 (11.1, 15.5) |
|  | Another identity/Multiracial | 15.8 (12.8, 18.9) | 20.7 (17.0, 24.4) |
|  | Missing | 3.0 (2.2, 3.7) | **-** |
| Mothers Education | High school or less | 14.1 (12.5, 15.6) | 17.6 (15.6, 19.5) |
|  | Some college | 19.9 (17.3, 22.6) | 23.9 (20.7, 27.0) |
|  | College or graduate | **17.5 (16.6, 18.5)** | **21.0 (19.8, 22.2)** |
|  | Missing | 7.5 (6.7, 8.4) | **-** |
| Have a physical or emotional disability | Yes | **18.0 (16.9, 19.1)** | **22.2 (20.9, 23.4)** |
|  | No | **15.0 (13.4, 16.5)** | **18.5 (16.6, 20.4)** |
|  | Missing | 0.5 (0.1, 0.8) | **-** |

Bold indicates that the 95% confidence intervals for prevalence estimates do not overlap.
